# Advances in the Phytochemical Characterisation and Bioactivities of *Salvia aurea* L. Essential Oil

**DOI:** 10.3390/plants12061247

**Published:** 2023-03-09

**Authors:** Jorge Miguel Alves-Silva, Delia Maccioni, Emma Cocco, Maria José Gonçalves, Silvia Porcedda, Alessandra Piras, Maria Teresa Cruz, Lígia Salgueiro, Andrea Maxia

**Affiliations:** 1 Institute for Clinical and Biomedical Research, Health Sciences Campus, University of Coimbra, Azinhaga de S. Comba, 3000-548 Coimbra, Portugal; 2 Faculty of Pharmacy, Health Sciences Campus, University of Coimbra, Azinhaga de S. Comba, 3000-548 Coimbra, Portugal; 3 Laboratory of Plant Biology and Pharmaceutical Botany, Department of Life and Environmental Sciences, University of Cagliari, Viale Sant’Ignazio 13, 09123 Cagliari, Italy; 4 Chemical Process Engineering and Forest Products Research Centre, Department of Chemical Engineering, Faculty of Sciences and Technology, University of Coimbra, 3000-548 Coimbra, Portugal; 5 Department of Chemical and Geological Sciences, University of Cagliari, Cittadella Universitaria, 09042 Monserrato, Italy; 6 Center for Neuroscience and Cell Biology, Faculty of Medicine, University of Coimbra, Rua Larga, 3004-504 Coimbra, Portugal

**Keywords:** antifungal, anti-inflammatory, senescence, wound healing, sage, *Salvia aurea* L., essential oil

## Abstract

The *Salvia* L. genus (Lamiaceae) is largely used in the pharmaceutical and food industry. Several species of biological relevance are extensively employed in traditional medicine, including *Salvia aurea* L. (syn. S. *africana-lutea* L.), which is used as a traditional skin disinfectant and in wounds as a healing remedy; nevertheless, these properties have not been validated yet. The aim of the present study is to characterise *S. aurea* essential oil (EO), unveiling its chemical composition and validating its biological properties. The EO was obtained by hydrodistillation and subsequently analysed by GC-FID and GC-MS. Different biological activities were assessed: the antifungal effect on dermatophytes and yeasts and the anti-inflammatory potential by evaluating nitric oxide (NO) production and COX-2 and iNOS protein levels. Wound-healing properties were assessed using the scratch-healing test, and the anti-aging capacity was estimated through the senescence-associated beta-galactosidase activity. *S. aurea* EO is mainly characterised by 1,8-cineole (16.7%), β-pinene (11.9%), cis-thujone (10.5%), camphor (9.5%), and (E)-caryophyllene (9.3%). The results showed an effective inhibition of the growth of dermatophytes. Furthermore, it significantly reduced protein levels of iNOS/COX-2 and simultaneously NO release. Additionally, the EO exhibited anti-senescence potential and enhanced wound healing. Overall, this study highlights the remarkable pharmacological properties of *Salvia aurea* EO, which should be further explored in order to develop innovative, sustainable, and environmentally friendly skin products.

## 1. Introduction

Medicinal plants have several organoleptic characteristics that contribute to their high value in the pharmaceutical, nutraceutical, cosmetic, and food industries. Their secondary metabolites are emerging as potential candidates for new anti-inflammatory and anti-fungal drugs [1,2,3]. Among others, aromatic species from the Lamiaceae family are well known for their richness in essential oils, demonstrating a wide range of biological effects, including, in particular, anti-inflammatory and antifungal [4,5,6,7,8]. One of the largest and most important genera of this family is the genus *Salvia* L., which comprises nearly 1000 species [9]. Some of these are cultivated worldwide for their culinary use as well as for medicinal purposes; in fact, they have long been employed in folk medicine [10,11]. Many species show important biological properties, including antibacterial, antifungal, antioxidant, anti-inflammatory, anticancer, hypoglycemic, and antidementia effects [12,13].

Although this genus has been extensively studied, there is still a great interest in those species that can be cultivated (domesticated) and used for health-promoting properties. In fact, it is well known that secondary metabolites are the result of plant/environmental interactions. Therefore, studies on the domesticated population can provide relevant information on whether secondary metabolites and their related biological properties vary from the native population. Among these, *Salvia aurea* L. (syn. *S. africana-lutea* L.) is an important aromatic species that is used for medicinal purposes in the Western Cape (South Africa) and is cultivated in many parts of the world as an aromatic/medicinal plant as well as ornamental (urban furniture). This species is a grey-green branched aromatic shrub that grows to about 2 m, with petiolate leaves that accumulate its essential oil in glandular trichomes and presents characteristically large and golden-brown flowers [14]. It is native to South Africa, where its geographical distribution extends westwards from the Cape Peninsula towards Namaqualand as well as eastwards from the Cape in the direction of Port Alfred in the Eastern Cape Province. It is often called either “beach sage, sandsalie”, which is typical of the dune environment, or “brown dune sage” for the characteristic colour of its flowers [15,16].

*S. aurea* is known for its several uses in traditional medicine, notably as an anti-inflammatory and medicinal wash for the treatment of wounds and skin affections. Furthermore, this species is also used for the healing of bronchitis and other respiratory ailments reinforcing its antimicrobial properties [17,18].

Dermatophytes, particularly those of *Trichophyton*, *Epidermophyton,* and *Microsporum*, are frequently associated with superficial mycoses [19]; however, immunocompromised individuals can be affected by invasive infections [20].

During these infections, fungal epitopes [21,22] bind to *Toll*-like receptors (TLRs), triggering the activation of a pro-inflammatory state. One of the most significant pathways associated with this condition is the nuclear factor kappa B (NF-κB) pathway, which involves the secretion of inflammatory cytokines and the synthesis of pro-inflammatory enzymes, for example, the inducible Nitric Oxide Synthase (iNOS) and the Cycloxygenase-2 (COX-2) [23]. These enzymes are responsible for nitric oxide (NO) and prostanoids production, respectively [24,25].

Furthermore, dermatophytes often lead to skin lesions [26]. Therefore, wound healing is needed to regenerate the damaged tissue; otherwise, the wound becomes chronic [27].

Fungal infections are still an unmet clinical need since the etiological agents are often associated with resistance to therapy and lead to relapses, while conventional drugs are widely associated with adverse effects [28]. Moreover, several fungal strains are known to be resistant to current antifungal agents [29,30]. Anti-inflammatory drugs have also been linked to several adverse effects [31]. Therefore, it is crucial to develop new strategies that, in addition to treating fungal infections, can also inhibit the pro-inflammatory state that is evoked during infection.

Given its traditional uses, it is likely that *S. aurea* might have concurrent antifungal and anti-inflammatory activities. However, these properties have been tested by a limited number of studies on a narrow range of microorganisms [32,33,34]. Furthermore, to the best of our knowledge, no studies have reported the wound-healing properties of *S. aurea* so far. Therefore, this work aims to characterize the essential oil of *S. aurea* (sandsalie) when domesticated in a coastal area of the island of Sardinia. Considering the oil variation with regard to season and locality, this territory was chosen for its similarities with the South African Fynbos ecoregion, *S. aurea* native territory [35,36]. In addition, we validated some of the traditional uses ascribed to this species, particularly those related to antimicrobial, anti-inflammatory, and wound healing uses. Anti-senescence effects were also disclosed to further promote interest in this plant.

## 2. Results

### 2.1. Chemical Composition of S. aurea Essential Oil

*S. aurea* leaves were subjected to hydrodistillation, obtaining the essential oil (EO) with 0.54% (*w*/*w*) yield. In Table 1, EO-identified compounds and their relative percentages are listed by their elution order. Thirty-six compounds were identified, for a total of 98.8%.

Furthermore, 47.4% of the compounds are oxygenated monoterpenes, 28.8% hydrocarbon monoterpenes, 19.8% hydrocarbon sesquiterpenes, and 2.8% oxygenated sesquiterpenes.

Chemical analysis revealed that *S. aurea* oil’s most representative components were 1,8 cineole (16.7%), β-pinene (11.9%), cis-thujone (10.5%), camphor (9.5%), (E)-caryophyllene (9.3%), trans-thujone (6.9%), α-pinene (4.7%), camphene (3.9%), and α-humulene (3.0%).

### 2.2. Antifungal Effect of Salvia aurea

The macrodilution broth method was used to assess the antifungal activity, as reported in Table 2. *Trichophyton mentagrophytes*, *T. rubrum,* and *Epidermophyton floccosum* are the most sensitive strains to EO activity. Specifically, EO showed a fungicidal effect for *T. mentagrophytes* and *E. floccosum*.

### 2.3. Anti-Inflammatory Potential of S. aurea

**Figure 1:**
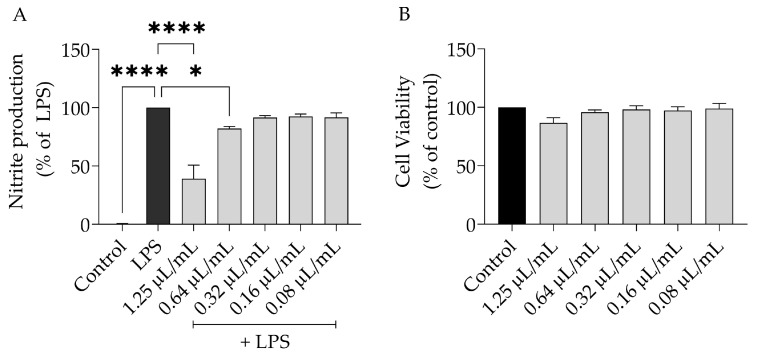
Effect of *S. aurea* EO on nitrite production. In (**A**), the tests were performed on LPS-stimulated macrophages (measurement after 1 h of pre-treatment with EO and subsequently 24 h of stimulation with LPS). (**B**) Represents the cell viability after 24 h of EO treatment. Results show the mean ± SEM. Statistical significance (* *p* < 0.05, **** *p* < 0.0001) was determined by either LPS or control comparison, through one-way ANOVA and consequent Dunnett’s multiple comparison test.

To assess the anti-inflammatory potential of *S. aurea,* we resorted to the lipopolysaccharide (LPS)-stimulated macrophages model. As expected, in the presence of LPS, the macrophages released NO to the medium, where it was detected as a nitrate ([NO] = 43.03 ± 7.74 µM). Interestingly, *S. aurea* EO reduced the NO release evoked by LPS dose-dependently (Figure 1A, IC_50_ = 1.07 µL/mL). The dose of 1.25 µL/mL was selected to unveil the underlying mechanisms as it inhibited NO production by more than 50%, was devoid of toxicity (Figure 1B), and was a dose that inhibited the growth of dermatophytes.

**Figure 2:**
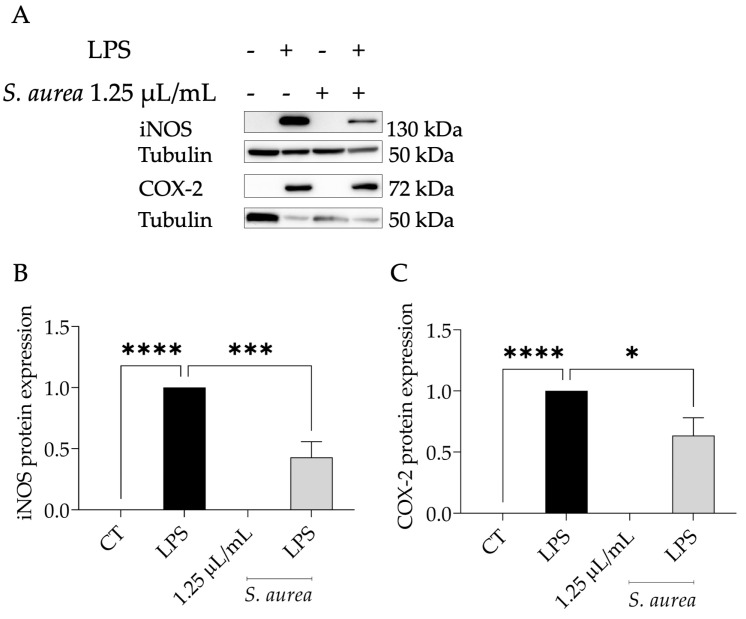
Effect of *S. aurea* EO on iNOS and COX-2 levels. (**A**) Shows representative blots. The positive (+) and negative (-) signals represent the presence or absence of the condition in the row. (**B**) Focuses on the semi-quantitative analysis of iNOS protein levels. Panel (**C**) focuses on the semi-quantitative analysis of COX-2 protein levels. Tubulin content was used to normalize the protein levels of iNOS and COX-2. Results show the mean +/- SEM. Statistical significance (* *p* < 0.05, *** *p* < 0.001, **** *p* < 0.0001) by either LPS or a control (CT) comparison was determined after one-way ANOVA test and consequent Dunnett’s multiple comparison test.

The LPS binding to the Toll-like receptor 4 (TLR4) triggers NF-κB nuclear translocation, which consequently causes the expression of inducible nitric oxide synthase (*Nos2*) and cycloxygenase-2 (*Ptgs2*) [39]. Then, we evaluated whether the EO could potentially regulate the levels of these proteins. As expected, in LPS-treated macrophages, an increase in the protein levels of both iNOS and COX-2 was observed (Figure 2A–C). The presence of *S. aurea* essential oil at the dose of 1.25 µL/mL could lead to a significant decrease in the protein levels of both proteins, especially iNOS. These results suggest that the essential oil might modulate the NF-κB pathway.

### 2.4. Wound Healing Properties of S. aurea Essential Oil

**Figure 3:**
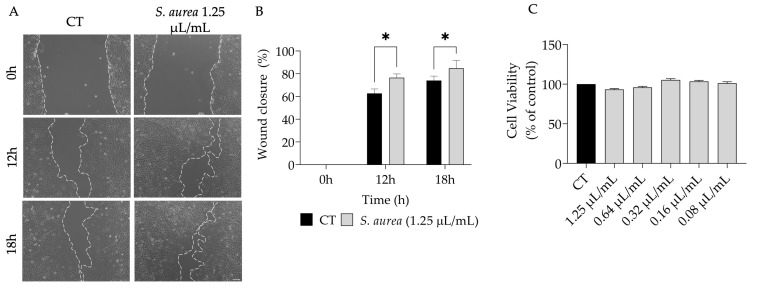
Effect of *S. aurea* essential oil on wound healing. (**A**) Shows a representative phase-contrast image of NIH 3T3 fibroblasts, respectively, in the absence (CT) and presence of *S. aurea* EO (1.25 µL/mL) at 0 h, 12 h, and 18 h post-scratch. (**B**) Expresses the percentage of a closed “wound”. (**C**) Shows the effect of *S. aurea* EO on NIH 3T3 fibroblast viability. Results show the mean ± SEM. Statistical significance (* *p* < 0.05) by control (CT) comparison was established after two-way ANOVA test followed by Sydák’s multiple comparison test or one-way ANOVA followed by Dunnett’s multiple comparison test.

Considering the uses of *S. aurea* for wound treatment [18], we hypothesized that the essential oil might enhance wound healing. Indeed, our results show that the EO promoted cell migration (Figure 3A,B) without affecting cell viability (Figure 3C).

### 2.5. Anti-Senescence Potential of S. aurea Essential Oil

**Figure 4:**
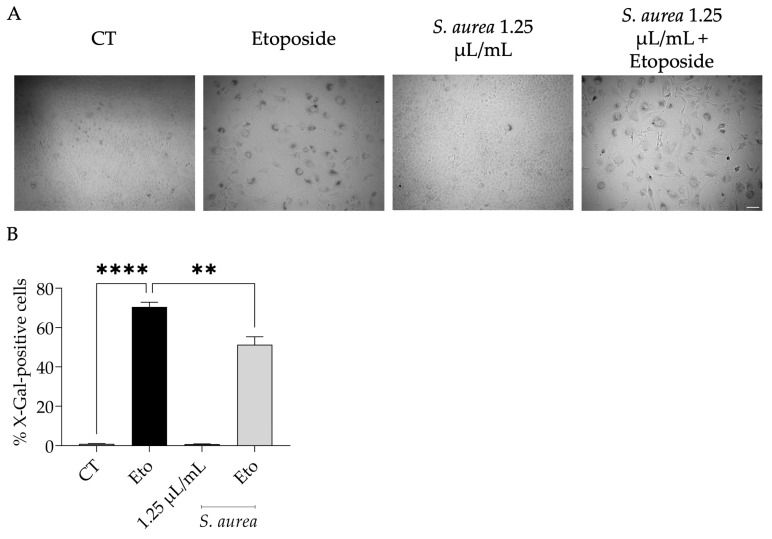
Effect of *S. aurea* EO on cell senescence. (**A**) Shows representative phase-contrast images of NIH 3T3 fibroblasts subjected to etoposide treatment (24 h etoposide incubation followed by 72 h of recovery period) in the absence (CT) or presence of *S. aurea* EO. (**B**) Expresses the percentage of X-gal-positive cells. Results show the mean ± SEM. Statistical significance ( ** *p* < 0.01, **** *p* < 0.0001) by control (CT) or etoposide (Eto) comparison was assessed after one-way ANOVA test and consequent Dunnett’s multiple comparison test.

Keeping in mind that the traditional uses ascribed to *S. aurea* are associated with the treatment of age-related diseases [40], we assessed the effect on cell senescence. Resorting to the etoposide-induced cell senescence, we observed senescence-associated β-galactosidase activity (Figure 4A,B). Interestingly, when the essential oil was added in the recovery phase, this feature was reduced, suggesting that the essential oil appeared to prevent cell senescence.

## 3. Discussion

This study provides the essential oil profile of *S. aurea* domesticated on the island of Sardinia. We reported that its EO was characterized by 47.4% of oxygenated monoterpenes, mainly 1,8-cineole, cis/trans-thujone and camphor, 28.8% of hydrocarbon monoterpenes, mainly β-pinene, and 19.8% of hydrocarbon sesquiterpenes, with (E)-caryophyllene, and α-humulene, while only 2.8% of oxygenated sesquiterpenes.

When compared with the literature, a clear qualitative and quantitative difference in native *S. aurea* EOs composition emerges. In particular, the major components reported were α-pinene, myrcene, o-cymene, spathulenol, and α-eudesmol [32]. Moreover, these alterations are reflected in the relative percentages of the main classes of compounds. In fact, here, we found a substantial decrease in oxygenated sesquiterpenes (2.8%), whereas they accounted for 18.9% and 41.7% in two different studies of the Western Cape region [33,41].

However, this chemical difference was not unexpected as, according to some authors, it depends not only on genetic characteristics but also on a plant’s life cycle, growing season, harvest season, soil properties, and stress agents [32,41].

In particular, with regard to *S. aurea,* a consistent chemical diversity was found according to seasonality and harvest territory [32,41]. In fact, Lim Ah Tock et al., 2020, considered populations of the Western Cape area and evidenced a high degree of intraspecific variability, accounting for 47.8% [32,33,41]. Furthermore, they analysed the HCA dendrogram of these populations, where a clear cluster separation appeared in *S. aurea*, to be responsible for the substantial chemical variation [41].

In addition to the mentioned factors that impact EO chemical composition, it is known that post-harvest treatment, storage conditions, and extraction methods also contribute to the chemical variability [42,43]. With this in mind, the differences that were observed here might be attributed to any of these factors.

The reported biological activities confirm the traditional uses attributes of *S. aurea*, particularly those associated with inflammatory and microbial infections.

We report that the EO inhibited the growth of dermatophytes, thus validating the use ascribed for the treatment of skin ailments [18]. The *S. aurea* essential oil is known to be effective against *Brevibacterium* [44], *Staphylococcus aureus*, *Klebsiella pneumoniae, Bacillus cereus,* and *Escherichia coli* [32,33]. Other studies reported antimicrobial activities for non-volatile extracts [14,45,46]. Regarding the major compounds found in *S. aurea* EO, it was reported that 1,8-cineole presented weak activity against the tested dermatophytes [47]. The inhibition of plant pathogens was also reported for 1,8-cineole [48,49,50,51]. The antimicrobial potential of β-pinene was also widely reported against several yeasts and filamentous fungi, including *Trichophyton* spp. [52,53,54,55,56]. *Cis*-thujone, a relevant compound in this essential oil, also exerted antimicrobial activity [57,58]. On the other hand, weak antimicrobial activity was reported for the camphor [59,60,61]. The sesquiterpene β-caryophyllene exerted antimicrobial activity against different fungi and bacteria [3,62,63,64]. These results suggest that β-pinene, *cis*-thujone, and β-caryophyllene might be major contributors to the activity reported for *S. aurea*.

Due to the traditional use of *S. aurea* in the treatment of inflammatory-related ailments [18], we aimed to validate these effects. Indeed, our results show that the EO inhibited the production of NO in LPS-stimulated macrophages and concomitantly reduced the protein levels of iNOS and COX-2: two pro-inflammatory enzymes relevant to the inflammatory response. To the best of our knowledge, only one study addressed the anti-inflammatory potential of *S. aurea* by showing the inhibitory capacity of lipoxygenase-5 activity [33]. Regarding isolated compounds, several studies reported the anti-inflammatory activity of 1,8-cineole [65,66,67,68,69,70], including clinical trials [71]. Furthermore, it is known that this compound inhibits COX-2 activity [66] and prevents the nuclear translocation of NF-κB [65,72]. β-pinene significantly decreased NO production in both LPS-stimulated macrophages [73] and IL-1β-activated chondrocytes [74]. It also showed a chemotaxis inhibition in neutrophils [75]. Camphor’s anti-inflammatory potential is widely reported using both in vivo and in vitro models [76,77,78]. β-Caryophyllene also exerted anti-inflammatory effects in colitis [79], skin wound excisions [80], and sepsis [81] in animal models. Other in vitro and in vivo approaches highlight the anti-inflammatory action of this sesquiterpene [82,83]. The results suggest that the activity observed in *S. aurea* could be due to the presence of these compounds, which might act synergistically.

We reported that the essential oil from *S. aurea* promoted wound healing, which validated its uses for the treatment of open wounds. To the best of our knowledge, no studies have reported the wound-healing properties of *S. aurea*. Regarding the isolated compounds, 1,8-cineole promotes cell migration in wound models both in vivo and in vitro [70,84,85]. Furthermore, it was reported that essential oil from *Teucrium polium* subsp. *capitatum,* rich in β-pinene, increased wound healing [86]. The sesquiterpene β-caryophyllene also promoted wound healing in a skin wound excision model [80]. Nevertheless, other compounds found in lower amounts could contribute to the reported activity. Indeed, α-pinene promotes wound healing in the animal model of an open wound [84]. Considering that essential oils are complex mixtures, it is conceivable that the reported wound-healing properties might be attributed to the synergic effects of several compounds.

We report, for the first time, an anti-senescence effect for *S. aurea* essential oil. Indeed, cells treated with the essential oil presented a reduced number of senescence-associated β-galactosidase-positive cells. Regarding isolated compounds, the number of studies reporting their effect was scarce. Indeed, camphor prevents the increased activity of senescence-associated β-galactosidase [87]. On the other hand, 1,8-cineole, the major compound of this essential oil, induced cell senescence [88]. Considering the scarcity of studies, we can only hypothesise that the reported activity might be due mainly to the camphor compound; however, the effect of minor compounds cannot be discarded.

## 4. Materials and Methods

### 4.1. Plant Material

African *S. aurea* seeds used for this study were acquired from a specialist store in Italy. The seedlings were cultivated in the Laboratory of Plant Biology and Pharmaceutical Botany of the University of Cagliari (UNICA). After 5 weeks, they were placed into a “Planta Medica” greenhouse, according to the eco-physiological needs of the species. The plants were then collected after two years of growth in their blooming period. The aerial parts were immediately placed in an air-forced ventilation oven (FD 115, BINDER) until completely dried (when they reached a constant weight). A voucher specimen was deposited at Herbarium Karalitanum (CAG) of the University of Cagliari, Italy, with voucher number (6/23.8/V1).

### 4.2. Essential Oil Analysis

The essential oil was obtained by 3 *h* of hydrodistillation using a Clevenger-type apparatus, according to the European Pharmacopoeia guidelines [89]. The subsequent analysis by gas chromatography with flame ionization detection (GC-FID) and gas chromatography/mass spectrometry (GC-MS) were conducted according to [13]. Briefly, for the GC analysis, an HP 5 capillary column was used, with an experimental procedure of 82 min at different temperatures, respectively, from 60 °C to 246 °C at a 3 °C/min rate, which was then kept at 246 °C for 20 min. Helium (purity ≥ 99.9999%) was used as a carrier gas at a 1 mL/min of flow rate. A total of 1 μL of the diluted sample (1:100 in n-hexane, *w*/*w*) was injected by an autosampler with a 1:20 split ratio. Regarding the MS conditions, a 240 °C transfer line, 200 °C EI ion source, and 150 °C of the quadrupole temperature were used, with ionization energy of 70 eV and 3.2 scans s-1 at m/z of scan range (from 30 to 480). MSD ChemStation software (Agilent, rev. E.01.00.237, Santa Clara, CA, USA) was used to manage and elaborate chromatograms and mass spectra. Finally, the obtained compounds were identified by NIST02 and Adams libraries comparison [37,38]. The results were further cross-checked by comparing the compounds’ experimental retention index (RI) with the semi-polar phases that RI reported in the literature. Experimental RIs were determined using two standard mixes of n-alkanes as the reference (C8–C20 and C21–C40, respectively) with linear interpolation [90]. The percentages of the reported components were computed on GC peak areas with no FID response factor correction.

### 4.3. Antifungal Activity

Seven dermatophyte strains were tested for *S. aurea* EO antifungal activity. Respectively, three clinical strains were obtained from nail and skin isolation: *Epidermophyton floccosum* FF9, *Trichophyton mentagrophytes* FF7, and *Microsporum canis* FF1. While the remaining four dermatophyte strains belonged to the Colección Espanõla de Cultivos Tipo (CECT): *T. mentagrophytes* var. *interdigitale* CECT 2958, *T. rubrum* CECT 2794, *T. verrucosum* CECT 2992, and *M. gypseum* CECT 2908. All strains were cultured in Sabouraud dextrose agar (SDA) or Potato dextrose agar (PDA) prior to each test to ensure purity and viability.

Minimal inhibitory concentrations (MIC) and the minimum lethal concentration (MLC) of EO were performed according to the modifications suggested by the CLSI protocol for macrodilutions [91]. Briefly, EO was diluted in DMSO (5–0.32 µL/mL) and then added to the sterile test tubes. Inoculum was prepared by adjusting the turbidity to 0.5 McFarland and then diluted in RPMI-1640 without glutamine and with 3-(N-morpholino) propanesulfonic acid (MOPS) pH 7.0 to a concentration of 1–2 × 10^4^ CFU/mL, which was then added to the test tubes containing the EO. The tubes were then incubated for 7 days at 30 ºC. Afterward, tubes were assessed for fungal growth, and the lowest concentration where no growth was observed was considered the minimum inhibitory concentration (MIC). The lowest concentration where no growth was observed after plating the negative tubes in SDA for 7 days at 30 ºC was considered the minimum lethal concentration (MLC). A reference antifungal compound, fluconazole (Pfizer), was used to control the sensitivity of the tested microorganisms. The results were obtained from three independent experiments performed in duplicate, and the results were expressed as the mean. Negative and positive controls were also included, represented by a non-inoculated medium and inoculated medium with the maximum DMSO concentration (1%), respectively.

### 4.4. Anti-Inflammatory Activity

#### 4.4.1. Cell Culture

The mouse leukemic macrophage RAW 264.7 cell line, which belonged to the American Type Culture Collection (ATCC TIB-71), was cultured as previously reported by our group [92].

#### 4.4.2. Nitric Oxide Production

NO production was evaluated by assessing the concentration of nitrates in culture supernatants using the Griess reagent [93]. Cells (0.6 × 10^6^ cells/well) were cultured in 48-well culture plates. Macrophages were stabilized overnight, then pre-treated for 1 h with EO (0.08–1.25 μL/mL) diluted in DMSO and subsequently activated with 50 ng/mL of LPS for 24 h. LPS-stimulated macrophages and untreated macrophages were used as the positive and negative controls, respectively. The Griess reaction was carried out as previously described in our group [92]. DMSO at the highest concentration used (0.4%) had already been shown by our group to have no anti-inflammatory or cytotoxicity activity (data not shown).

#### 4.4.3. Expression of Pro-Inflammatory Proteins, iNOS and COX-2

RAW 264.7 cells (1.2 × 10^6^ cells/well) were cultured in 6-well plates and stabilized overnight. Then, these cells were subjected to 1 h of incubation with EO at a 1.25 µL/mL concentration, followed by 24 h of LPS activation (50 ng/mL). A negative control was constituted of the untreated cells, and a positive control was constituted of only LPS-treated cells. Cell lysates preparation followed the protocol previously performed by Zuzarte et al. [92]

Inducible nitric oxide synthase (iNOS) and cyclooxygenase-2 (COX-2) content were assessed by Western blot analysis as previously described [13]. For protein separation, an electrophoretic run with 10%(*v*/*v*) SDS-polyacrylamide gels at 130 V was performed for 1.5 h. The protein lines were consequently blotted to polyvinylidene fluoride membranes (previously activated with methanol) at 400 mA for 3 h. The membranes were then incubated for 1 h at room temperature with non-specific IgGs with 5% (*w*/*v*) skim milk in TBS-T. They were further incubated overnight at 4 °C with specific anti-iNOS (1:500; R & D Systems) or anti-COX-2 (1:5000; Abcam, Cambridge, UK) antibodies. Finally, they were washed for 30 min with TBS-T (10 min, 3 times) and incubated for 1 h at room temperature with secondary antibodies (1:40,000; Santa Cruz Biotechnology, Dallas, TX, USA) conjugated with horseradish peroxidase. The immunocomplexes detection was performed by a chemiluminescence scanner (Image Quant LAS 500, GE, Boston, MA, USA). Antibodies against tubulin (1:20,000; Sigma, St. Louis, MO, USA) were used as the loading control. ImageLab software version 6.1.0 (Bio-Rad Laboratories Inc., Hercules, CA, USA) was used for protein quantification.

### 4.5. Cell Migration

#### 4.5.1. Cell Culture

The mouse embryonic fibroblast cell line NIH 3T3 (ATCC CRL-1658) was cultured as previously described in [6].

#### 4.5.2. Cell Migration Assay

Cell migration was carried out using the scratch wound assay according to Martinotti et al. [94] with slight modifications, as previously reported [13]. Briefly, NIH 3T3 fibroblasts were seeded at 2.5 × 10^5^ cells/mL and allowed to reach confluency. Afterward, the wound was inflicted with a 200 µL pipette tip, and non-adherent cells were removed by washing with PBS pH 7.4. DMEM with 2% FBS with or without the EO (1.25 µL/mL) being added. The images were acquired at 0, 12, and 18 h post-scratch using a phase-contrast microscope, and the wound area was measured using ImageJ/Fiji software. The results presented were obtained using the following equation:

Wound closure (%) = (At = 0 h – At = x h)/At = 0 h × 100

where At = 0 h is the area of the wound 0 h after the scratch and At = x h is the area at 0 h, 12 h, and 18 h post-scratch.

### 4.6. Cell Viability

The effect of different concentrations of EO on the viability of macrophages and fibroblasts was assessed using the resazurin reduction assay, as previously reported [6].

### 4.7. Etoposide-Induced Senescence

Senescence was evaluated using etoposide as a senescence inducer, as reported elsewhere [95], with some modifications. Briefly, after 24 h of etoposide, the cells were further incubated for 72 h in the presence or absence (CT) of *S. aurea* EO. Beta-galactosidase was assessed using a commercially available kit according to the manufacturer’s protocol (#9860, Cell Signalling Technology Inc., Danvers, MA, USA). The distinct blue colour staining indicates beta-galactosidase activity. After colour developments, the wells were photographed for subsequent image analysis. ImageJ software was used for quantitative analysis by assessing the percentage of senescent cells.

### 4.8. Statistical Analysis

The experiments were performed at least in duplicate for three independent experiments. Mean values ± SEM (standard error of the mean) are presented in the results. Statistical significance for anti-inflammatory, cell viability, and senescence assays was evaluated by a one-way analysis of variance (ANOVA) and Dunnett’s post hoc test using GraphPad Prism version 9.3.0 (GraphPad Software, San Diego, CA, USA). While the statistical significance for cell migration assays was assessed using two-way ANOVA followed by Sydák’s multiple comparison tests, *p* values < 0.05 were accepted as statistically significant.

## 5. Conclusions

This work reinforces the beneficial effects usually attributed to *Salvia* spp. by validating some of the traditional uses ascribed to *S. aurea*. In addition, a singular chemical composition was disclosed with 1,8 cineole, β-pinene, *cis*-thujone, camphor, (E)-caryophyllene, *trans*-thujone, α-pinene, camphene, and α-humulene as major compounds. We herein report that the EO exerts antifungal, anti-inflammatory, and wound-healing effects, thus validating the traditional uses associated with this species for the treatment of skin infections, inflammation-related diseases, and wounds.

In addition, this study reports, for the first time, that this species was able to exert anti-senescence effects, thus further promoting interest in this species. Therefore, these results highlight the role of *S. aurea* in mitigating inflammation and skin-associated infections, thus reinforcing interest in dermo-cosmetics. While this study has shown that the production of high-value metabolites with relevant biological activities can be promoted through cultivation, an accurate chemical analysis of the cultivated plant is essential, considering the extreme variability of the chemical profile as a result of genetic and environmental factors (stress, soil properties, harvest season).

## Figures and Tables

**Table 1:** Chemical composition of *S. aurea* essential oil.

R_I_	R_I_ (Litt)	Compound	Area, %
925	921	tricyclene	0.1
927	924	α-thujene	1.0
934	932	α-pinene	4.7
951	946	camphene	3.9
973	969	sabinene	0.7
980	974	β-pinene	11.9
989	988	myrcene	2.1
1017	1014	α-terpinene	0.7
1024	1022	ortho-cymene	0.4
1029	1024	limonene	1.5
1033	1026	1,8-cineole	16.7
1044	1044	(E)-β-ocimene	0.3
1056	1054	γ-terpinene	1.2
1068	1065	cis-sabinene hydrate	0.2
1084	1086	terpinolene	0.3
1100	1095	linalool	0.3
1107	1101	cis-thujone	10.5
1117	1112	trans-thujone	6.9
1146	1141	camphor	9.5
1169	1165	borneol	2.7
1178	1174	terpinen-4-ol	0.4
1192	1186	α-terpineol	0.2
1343	1345	α-cubebene	0.5
1364	1373	α-ylangene	0.3
1371	1374	α-copaene	0.7
1415	1417	(E)-caryophyllene	9.3
1424	1430	β-copaene	0.6
1432	1439	aromadendrene	0.8
1449	1452	α-humulene	3.0
1469	1478	γ-muurolene	1.6
1486	1495	γ-amorphene	0.3
1493	1500	α-muurolene	0.3
1507	1513	γ-cadinene	0.5
1513	1522	δ-cadinene	1.8
1574	1582	caryophyllene oxide	0.4
1587	1592	viridiflorol	2.4
**Total identified**			**98.8**
**Hydrocarbon monoterpenes**			**28.8**
**Oxygenated monoterpenes**			**47.4**
**Hydrocarbon sesquiterpenes**			**19.8**
**Oxygenated sesquiterpenes**			**2.8**

R_I_, retention index determined on a HP-5ms fused silica column relative to a series of n-alkanes R_I_ (Litt), retention index reported from Adams library [37]. Compounds were identified by comparing their mass spectra (MS) and retention indices (RI) with those reported in Adams and NIST02 libraries [37,38].

**Table 2:** Minimum inhibitory (MIC) and lethal (MLC) concentrations of *S. aurea* essential oil against dermatophytes.

Strains	*S. aurea*		Fluconazole	
	MIC ^a^	MLC	MIC ^b^	MLC
*Trichophyton mentagrophytes* FF7	1.25	1.25	16–32	32–64
*T. rubrum* CECT 2794	1.25	2.5	16	64
*T. mentagrophytes var. interdigitale* CECT 2958	5	>5	128	≥128
*T. verrucosum* CECT 2992	5	>5	>128	>128
*Microsporum canis* FF1	5–2.5	>5	128	128
*M. gypseum* CECT 2908	5	>5	128	>128
*Epidermophyton floccosum* FF9	1.25	1.25	16	16

^a^ MIC and MLC are expressed in µL/mL (*v*/*v*); ^b^ MIC and MLC are expressed in µg/mL (*w*/*v*).

## Data Availability

Data will be available upon request.
